# A review of extended coronavirus disease 2019 (COVID-19) isolation duration among inpatients in a tertiary-care hospital—Iowa, 2020–2022

**DOI:** 10.1017/ice.2023.154

**Published:** 2024-01

**Authors:** Oluchi J. Abosi, Alexandra Trannel, Patrick Schwartzhoff, Mark Ackman, Barbara Zilles, Alexandre R. Marra, Angelique Dains, Toshio Naito, Jorge L. Salinas, Daniel J. Diekema, Beth Hanna, Jaime P. Murphy, Melanie Wellington, Karen Brust, Takaaki Kobayashi

**Affiliations:** 1 University of Iowa Hospitals & Clinics, Iowa City, Iowa, United States; 2 Hospital Israelita Albert Einstein, São Paulo, Brazil; 3 General Medicine, Juntendo University Hospital, Tokyo, Japan; 4 Stanford University, Stanford, California, United States

## Abstract

Of the 2,668 patients admitted with coronavirus disease 2019 (COVID-19), 4% underwent prolonged isolation for >20 days. Reasons for extended isolation were inconsistent with Centers for Disease Control and Prevention (CDC) guidelines in 25% of these patients and were questionable in 54% due to an ongoing critically ill condition at day 20 without CDC-defined immunocompromised status.

The Centers for Disease Control and Prevention (CDC) recommended placing patients with mild-to-moderate coronavirus disease 2019 (COVID-19) under transmission-based (ie, isolation) precautions for at least 10 days from onset of symptoms or up to 20 days if they had severe-to-critical illness. Since moderately and severely immunocompromised patients can shed viable viruses for prolonged periods of time regardless of disease severity, the duration of isolation could extend beyond 20 days.^1^ However, adherence to the extended isolation duration recommendation by the CDC has not been well studied in the United States. We describe the population admitted with a diagnosis of COVID-19 in our hospital isolated for >20 days as well as reasons for prolonged isolation duration.

## Methods

This retrospective observational study was performed between January 2020 and January 2022 at The University of Iowa Hospitals & Clinics (UIHC), an 860-bed academic medical center. We obtained *International Classification of Diseases, Tenth Revision* (ICD-10) codes for all patients admitted during the study period from the electronic medical record (EMR), and we identified inpatients with an ICD-10 code of U07.1 for COVID-19. Based on CDC isolation recommendations, we divided eligible participants into the following 3 groups: those with isolation duration ≤10 days (group 1), those with isolation duration >10 days but ≤20 days (group 2), and those with isolation >20 days (group 3).^1,3^ During the study period, UIHC policy required patients with suspected or confirmed COVID-19 to have an isolation order for airborne and contact precautions plus eye protection. We performed manual chart review for patients identified with an isolation duration of >20 days (group 3) to identify reasons for prolonged COVID-19 isolation. Reasons for extended COVID-19 isolation were divided into the following categories: (1) unclear, (2) clinically improving but persistently low oxygen requirement (≤4 L/min), (3) severe respiratory failure (requiring invasive or noninvasive mechanical ventilation, high-flow nasal canula, or oxygen requirement >4 L/min), (4) respiratory failure with shock (pressor requirement), and (5) moderately or severely immunocompromised. We then divided patients into the following 3 categories based on the congruency of isolation duration with CDC guidance: consistent, questionable, and inconsistent. We considered extended isolation >20 days as being consistent with public health guidance if “moderately and severely immunocompromised” status met the CDC definition.^1–4^ No clear public health guidance is available regarding isolation durations for those who are not immunocompromised but remain severely ill; thus, the category “questionable” was used if the reasons for extended isolation were either severe respiratory failure or respiratory failure and shock at day 20 of hospitalization. Inconsistent category included those in isolation for unclear reasons and those with improving respiratory symptoms but a persistently low oxygen requirement (≤4 L/min).

## Results

During the study period, we identified 2,668 patients admitted to UIHC with a COVID-19 diagnosis. Among them, 2,089 (78%) patients were in group 1, 466 (17%) were in group 2, and 113 (4%) were in group 3 (Table 1). Death during admission occurred in 9%, 27%, and 35% of patients, respectively (*P* < .001). Results of the manual chart review for 113 patients (group 3) are shown in Supplementary Table 1 (online). We confirmed that all 113 patients were considered to have active COVID-19 by treating providers. Most patients, 109 (96%), in this group were not vaccinated prior to the admission for COVID-19. Among the 113 patients, 107 (95%) were admitted to the ICU and 87 (77%) were intubated. Of 113 cases, 65 (57.5%) occurred in 2020, 48 (42.5%) occurred in 2021, and none occurred in 2022.

**Table 1.** Characteristics of Those With COVID-19 Diagnosis Stratified by Isolation Duration via Administrative Data (N = 2,668)

Characteristic	Duration of Isolation Categories			
	Group 1 (n = 2,089, 78%), No. (%)^a^	Group 2 (n = 466, 17%), No. (%)^a^	Group 3 (n = 113, 4%), No. (%)^a^	*P* Value
Isolation duration, d	≤10	>10 but ≤20	>20	
Isolation duration, mean d (SD)	4.4 (2.5)	14.9 (2.7)	25.0 (5.2)	<.0001
Isolation duration, range, d	1–10	11–20	21–54	
Interquartile range, d	2–6	12–16	22–27	
**Year of admission**				
2020	942 (45.1)	199 (42.7)	65 (57.5)	<.0001
2021	932 (44.6)	243 (52.1)	48 (42.5)	
2022	215 (10.3)	24 (5.2)	0	
Age, mean y (range)	49 (0–101)	58 (0–100)	54 (0–88)	<.0001
**Sex**				
Female	938 (45)	169 (36)	46 (41)	.003
Male	1,151(55)	297 (64)	67 (59)	
**Race**				
White	1,531 (73)	347 (74)	78 (69)	.30
African American/Black	243 (12)	38 (9)	13 (11)	
Hispanic/Latino of any race	223 (11)	53 (11)	17 (15)	
Multiracial	23 (1)	6 (1)	1 (1)	
Asian	25 (1)	8 (2)	3 (3)	
American Indian	7 (0.3)	5 (1)	0 (0)	
Native Hawaiian	4 (0.2)	1 (0.2)	1 (1)	
Declined	33 (2)	8 (2)	0	
Duration of isolation, mean d (SD)	4.4 (2.5)	14.88 (2.7)	25.0 (5.2)	<.0001
**Discharge disposition**				
Home or self care	1508 (72)	171 (37)	26 (23)	<.0001
Died	197 (9)	125 (27)	39 (35)	
Skilled-care facility	63 (3)	40 (9)	7 (6)	
Inpatient rehabilitation facility	47 (2)	44 (9)	18 (16)	

a Data are no. (%) unless otherwise specified.

Reasons for isolation that extended beyond 20 days were severe respiratory failure in 34 patients (30%), followed by respiratory failure with shock in 27 patients (24%), moderately or severely immunocompromised in 24 patients (21%), improving but persistently low oxygen needs in 15 patients (13%) and unclear reasons in 13 patients (12%) (Table 2). Reasons for prolonged isolation were questionable in 61 patients (54%) who were either still in severe respiratory failure or in shock. In 28 patients (25%), isolation was considered inconsistent with public health guidance due to either an unclear reason or minimal oxygen requirements but otherwise improved.

**Table 2.** Documented or Probable Reasons for Prolonged Isolation >20 Days

Consistency with CDC Isolation Guidelines	Reason	Total (N = 113), No. (%)
Inconsistent (N = 28)	Unclear reasons	13 (12)
	Persistent oxygen requirement (≤4 L/m)	15 (13)
Questionable (N = 61)	Respiratory failure requiring mechanical ventilation, noninvasive mechanical ventilation, or oxygen >4 L/m	34 (30)
	Respiratory failure and shock	27 (24)
Consistent (N = 24)	Moderately or severely immunocompromised	24 (21)
	Hematologic malignancy not on treatment	3
	Hematologic malignancy on treatment^a^	2
	Active cancer not on treatment	6
	Active cancer on treatment^a,^ ^b,^ ^c^	4
	Solid-organ transplant on treatment^a,^ ^b^	5
	Autoimmune disorders, and other medical illnesses on treatment^‡^	4

a Immunosuppressive agents used in treatment of malignancies, autoimmune disorders, and other medical illnesses.

b Steroid use of ≥20 mg prednisone or equivalent per day when administered for ≥2 weeks.

c Some patient received steroids in addition to other immunosuppressive agent.

## Discussion

Only 4% of patients admitted with COVID-19 were in isolation >20 days. Of 113 patients, 25% with an isolation duration of >20 days were placed in isolation longer than recommended. Among these 28 patients, 15 had prolonged isolation due to a persistently low oxygen requirement (≤4 L/min) on isolation day 20. The CDC has specified discontinuation of isolation after symptom improvement, but this is not well described. Some providers may have concerns about persistent viral RNA detection in samples undergoing real-time polymerase chain reaction testing, particularly with ongoing symptoms. They may have felt more comfortable continuing isolation until symptoms completely resolved. However, we observed longitudinal improvements in the CDC guideline applications. Better understanding of infection transmission and continued guideline dissemination efforts might have increased compliance. Additionally, we categorized 61 (54%) of 113 patients as questionable because they were still critically ill or had worsening severe respiratory failure with or without shock on isolation day 20 in the absence of a pre-existing immunocompromised status. Currently, no clear guidance is available regarding when to stop isolation for those who remain critically ill on isolation day 20. Clear evidence-based guidelines for COVID-19 isolation are needed, especially for immunocompetent but critically ill patients.

Prolonged isolation duration may have consequences that outweigh the benefits. For example, prolonged isolation precautions could decrease HCP contact with patients and their family, potentially leading to worse clinical outcomes.^5,6^ Psychological impacts, such as an increase in symptoms of depression and anxiety, have been reported in patients kept in isolation for extended periods.^7,8^ Additionally, prolonged isolation requires increased use of disposable PPE such as surgical masks, gowns, and gloves and the associated costs are notable.^9^ Furthermore, prolonged isolation for COVID-19 patients affects staffing and bed availability. Discontinuing unneeded isolation promptly may help alleviate bed availability constraints by discharging noninfectious patients to nursing homes or rehabilitation facilities sooner.

This study had several limitations. It was a retrospective, single-center study, and these findings may not be generalizable to other settings. We did not perform manual chart review for the groups 1 and 2 and thus did not investigate appropriateness of isolation for duration up to 20 days. Some patients were admitted more than once. Although all the patients in group 3 were considered to have active COVID-19 by manual chart review, some might have had a previous COVID-19 case, and we did not use cycle threshold value. During our manual chart review, we focused on EMR documentation around hospitalization day 20. However, this procedure might not correlate with the CDC guidance of 20 days from symptom onset given that the patients do not present to the hospital until several days after infection.

In conclusion, prolonged isolation occurred for 4% of patients admitted with COVID-19. Approximately one-fourth with an isolation duration >20 days were isolated for reasons inconsistent with CDC guidance, and another one-half were isolated for questionable reasons for prolonged isolation. Healthcare facilities should monitor isolation precautions adherence for COVID-19 patients. Further studies are needed to investigate SARS-CoV-2 transmissibility >20 days from symptom onset in patients who remain critically ill but are not immunocompromised. In cases in which patients continue to require oxygen supplementation, the CDC needs to define the term improvement of symptoms.
